# Going beyond compliance to build on the Researcher Development Concordat

**DOI:** 10.7554/eLife.110126

**Published:** 2026-01-15

**Authors:** Robert AR Drake, Laura A Gray, Cathal Rogers, Brianna Vandrey, Joan Chang

**Affiliations:** 1 School of Psychology and Neuroscience, University of Bristol Bristol United Kingdom; 2 School of Medicine and Population Health, University of Sheffield Sheffield United Kingdom; 3 University of Manchester Manchester United Kingdom; 4 School of Psychology & Neuroscience, University of Glasgow Glasgow United Kingdom; 5 School of Biological Sciences, University of Manchester Manchester United Kingdom

**Keywords:** research culture, early-career researchers, careers in science, Researcher Development Concordat, None

## Abstract

An agreement between universities, research institutes and funders to support the career development of researchers in the UK has led to improvements in research culture since 2019, but there is still more to do.

## Introduction

Research culture has become a central theme in discussions about the sustainability and quality of research. Increasingly, funders, institutions, and policymakers recognise that the quality and integrity of research are shaped by the conditions in which it is undertaken. It is now widely understood that issues related to lack of job security, recognition, equity, and wellbeing are not just workforce concerns, they are central to innovation and integrity in research.

Postdoctoral researchers constitute the backbone of the research enterprise in most countries, driving discovery, training PhD students, and supporting innovation both inside and outside of academic institutions. However, postdocs also suffer from a lack of job security and bleak long-term job prospects. In the UK, for example, around 70% of early-career researchers would like to pursue an academic career (Vitae, 2023), but longitudinal data show that only around 30% remain in academic research three years after completing a PhD (Hancock, 2023), and fewer than 3.5% secure permanent academic posts (Royal Society, 2010). Among current postdoctoral staff, approximately 70% are employed on fixed-term contracts – half of which are two years or shorter – illustrating the pervasive insecurity of academic employment (Vitae, 2025).

## The Researcher Development Concordat

Published in 2019, the Researcher Development Concordat provides a national framework for addressing challenges related to research culture in the UK (https://researcherdevelopmentconcordat.ac.uk/). This article has its origins in a round-table discussion convened by the Centre for Postdoctoral Development in Infrastructure, Cities and Energy in 2023 to identify barriers, priorities, and best practices in implementing Concordat ahead of that year’s National Postdoc Conference. Although we will focus on research culture in the UK, many of the problems and issues we will discuss are relevant to postdoctoral researchers across the globe.

The Researcher Development Concordat is built around three core principles: Environment and Culture; Employment; and Professional and Career Development. Under the Environment and Culture principle, institutions and funders are expected to foster positive, inclusive, and supportive research settings that uphold the highest standards of integrity. This includes promoting equality, diversity, and inclusion; tackling bullying and harassment; and supporting wellbeing and work-life balance. Notably, there should be transparent communication of institutional policies and expectations.

The Employment principle emphasises that researchers must be recognised and valued as professionals, with fair and transparent recruitment and progression practices. Employers should reduce reliance on short-term contracts, explore mechanisms to enhance job security, and provide clear routes for career advancement. Managers of researchers should be properly trained in leadership, equality, wellbeing, and integrity, to ensure that good people management underpins all employment practices.

Finally, the Professional and Career Development principle focuses on helping researchers to thrive within and beyond academia. Institutions should guarantee researchers a minimum of ten days per year for professional development activities, and ensure they have access to mentoring, skills training, and meaningful annual career reviews. Development opportunities should prepare researchers for a wide range of career paths, and their engagement in such activities should be supported and recognised.

Collectively, these three principles aim to create a research system that values its people, nurtures their growth, and sustains excellence through an equitable and empowering culture.

## What is working well?

The Concordat has clarified expectations for funders, institutions, managers, and researchers around shared responsibility for researcher development. This is particularly important for large institutions with a high turnover of people, where it can be challenging to maintain the focus and commitment required to change research culture. Signatories of the Concordat have developed public-facing action plans that outline their initiatives to meet the expectations of the Concordat, such as: automatic conversion to open-ended contracts beyond a minimum service period; improving the transparency of promotions criteria and using narrative CVs to capture performance more holistically; and increasing the resources for researcher development. Universities have also introduced Concordat Champion roles, typically held by tenured members of the faculty, to provide continuity in the local implementation of policies, and to act as bridges between researchers and management. This structure helps maintain momentum, evaluate progress locally, and adapt actions to evolving needs.

Recognizing postdoctoral researchers as valued professionals – as called for by the Concordat – has encouraged universities to include their perspectives when developing strategy, and this has had a positive impact on research culture. Many universities have convened committees that include professional services, HR, management, and researchers to implement the Concordat. Postdocs are also gaining a stronger voice in governance: some now sit on departmental or faculty committees, meet regularly with senior colleagues, and contribute to discussions beyond researcher support. The inclusion of postdocs in these committees enriches institutional decision-making while also giving researchers greater ownership of their working environment.

Funders, including UK Research and Innovation (UKRI) and the Wellcome Trust, have also engaged directly with postdocs through town hall meetings and conducted in-depth analysis of research culture in the UK (UKRI, 2024; Wellcome, 2020). This has led to commitments for improving research culture through direct funding, and the inclusion of factors related to research culture in the criteria for certain research grants (Wellcome, 2025).

Research culture and the development of people is now more explicitly embedded within the Research Excellence Framework (REF), the mechanism through which UK Universities are assessed, and public funding for research is allocated. In December 2025, following a three month pause, Research England confirmed that a Strategy, People, and Research Environment (SPRE) element will contribute 20% of institutions scores in the next REF exercise (REF, 2023). Although this represents a reduction from the originally proposed 25%, the inclusion of SPRE places people firmly at the centre of the assessment, requiring institutions to evidence the strategies and practices that underpin excellence in research culture and the ongoing support and development of research staff (REF, 2025a).

However, in absence of robust and objective measures of research culture, capable of evaluating both quality and progress within and across institutions, universities and REF assessment panels are likely to face significant challenges in demonstrating, interpreting and comparing performance in this domain. A recent report commissioned as part of the preparations for the next REF seeks to address these challenges by developing a shared framework, clearer definitions, and a more consistent set of indicators to support assessment of research culture across institutions (REF, 2025b; REF, 2025c). Nevertheless, reliance on narrative evidence and locally driven implementation means progress remains vulnerable to staff turnover, limited institutional memory, and the absence of longitudinal, objective measures.

## What could be improved?

While the Concordat has made real progress, several barriers have limited its impact.

### Professional development is undervalued and under-provided

Discovery research does not always produce immediate outputs, yet careers depend on them. Crucially, discovery research drives innovation when properly engaged with industry, public and governance. However, in a publish-or-perish culture, professional development and engagement activities are routinely sidelined. In 2023 a survey found that only about 16% of researchers take up the Concordat’s entitlement to 10 days of development each year, and nearly half report insufficient time to build leadership skills or their research identity (Vitae, 2023). An update in 2025 suggests little improvement, with 25% of responders spending less that one day per year on training or professional development (Vitae, 2025).

Simple, low-cost steps could help. Protect time by explicitly allocating development days; promote role models by showcasing success stories; broaden opportunities through small grants or networking; and provide in-house training. Crucially, embedding professional development into appraisal, promotion, and grant criteria would send a stronger message: it is not a distraction from research, but a core part of building sustainable careers within and beyond academia. Expanding criteria is welcomed, but should require funders and, importantly, institutions to embed training and resources directly into research roles. Many institutions have expanded criteria for promotion but may not have provided the appropriate training and resources, including time, to complete these activities. For example, the move to narrative CVs better captures performance and outcomes but without proper training in both the writing and the assessment of them, it can create an unfair system.

### Concordat Action Plans lack focus on the issues that affect postdocs most

While Concordat Action Plans have delivered welcome improvements, they do not fully address the structural issues shaping postdoctoral careers. Precarity remains the defining challenge, driven by serial fixed-term contracts, unclear career progression routes, and limited protection against job insecurity. A survey in 2023 found that half of research staff have held two or more fixed-term contracts at their institution (Vitae, 2023). Moreover, although two-thirds are contracted for 80–100% research time, only half actually achieve this, because many postdocs shoulder substantial teaching, supervision and administrative responsibilities that the system rarely acknowledges.

A central concern is the lack of clear, fair promotion pathways. For many postdoctoral researchers, progression depends less on merit than on whether funding exists to cover an increase in their salary. Indeed, according to the 2023 survey, only 33% believe that promotion is merit-based, so they don’t know if time spent on teaching, stakeholder engagement or their own professional development will provide a return on investment. Serial fixed-term contracts compound this problem; many postdocs move from grant to grant within the same institution without accruing the long-service rights that underpin equitable promotion processes.

Fixed-term contracts create financial and personal instability by offering reduced legal protection and preventing continuity of service. They limit access to long-service rights such as redundancy pay, enhanced parental leave, and incremental pay progression, and they undermine financial security, mortgages, loans, and even rental agreements often require evidence of ongoing employment beyond a fixed term.

An alternative model is the open-ended, funding-contingent contract, used in sectors reliant on external income such as charities, NGOs, the creative industries, government, and tech start-ups. These are permanent contracts in which employment continues for as long as funding is available. Crucially, they confer the same legal protections as other permanent roles, including unfair dismissal rights, formal redundancy processes, redeployment obligations, and compensation.

Despite this, such contracts are rare in academia: in 2025, only 13% of researchers were employed on open-ended contracts, whereas 64% were on fixed-term contracts, with half of these contracts lasting less than two years (Vitae, 2025). This is not a necessity but a policy choice, one that undermines workforce stability, limits diversity and retention, and weakens research culture.

To address these challenges, the use of fixed term contracts should be limited to instances of necessity. Promotion procedures should also be transparent with criteria that value a broader range of contributions, and contracts should include protected time and resources for these contributions. Salary headroom for progression should be built into grants by default, with employers willing to bridge temporary gaps. Where this is not feasible, institutions should offset relocation costs, visa fees and similar burdens that are often associated with fixed-term roles. These changes would give postdocs a clearer, fairer and more secure path forward, which would encourage forward planning, develop individuals and retain talent.

### Visibility and resources

It is critical that postdocs understand what the Concordat entitles them to. This awareness can empower them to advocate for appropriate support and help sustain the development of initiatives that are vulnerable to high staff turnover. Introducing the Concordat and Concordat Action Plans at staff inductions, using postdoctoral representatives to cascade information, and providing regular updates on Concordat Action Plan progress could improve visibility. Some institutions also suggest designating time for Concordat activities, such as a set week each year (such as National Postdoc Appreciation Week) or recurring days. Resourcing is equally critical, and funding for research culture does exist; for example, Research England has an Enhancing Research Culture fund offering opportunities to support the delivery of Concordat Action Plans. We recognise the higher education sector in the UK is under significant financial pressure but it is important that universities continue to invest in both research culture and the development of the individuals. If Institutions wish to protect their global position in science and innovation they must develop innovative approaches to sustaining fundamental research and the culture that supports it.

### Data for accountability and improvement

A central challenge is understanding what is genuinely improving – and why. Stronger data collection and transparent reporting would allow institutions to demonstrate progress, refine their approaches, and make researcher development a visible, valued part of university life. The need for objective, comparable and longitudinal metrics to track research culture is widely recognised (Hancock et al., 2019), and national initiatives such as the People, Culture and Environment indicators project aim to address this by 2026 (REF, 2025b). Crucially, such reporting can build on existing systems and need not be burdensome.

Robust insight into the workforce is essential. Basic information on researcher numbers, contract types and salary grades, alongside data on career progression and promotions, fellowship outcomes, faculty appointments and destinations of leavers, would show whether current policies are supporting researchers effectively and where bottlenecks persist. Reporting on professional development activity (e.g., researcher roles on policy or governance committees) and engagement with Concordat Action Plan initiatives (such as uptake of the 10 day entitlement, secondments and training) would help staff development teams target resources and direct funding toward the most impactful interventions. Publishing these data, together with evidence of how research culture is recognised and rewarded, such as funding for activities, institutional awards, and how promotion criteria capture these contributions, would offer a coherent picture of institutional practice.

Collecting and sharing this information brings multiple benefits. Internally, it enables Concordat Champions and committees to allocate resources strategically and evaluate impact. Externally, it provides funders with tangible evidence of commitment and highlights effective models for supporting researchers and research culture. Importantly, it also empowers postdocs to make informed decisions about where to work. More broadly, transparent reporting would shift the Concordat from a perceived compliance exercise to an evidence-driven framework for meaningful cultural change.

## Conclusion

The Concordat has created meaningful momentum across the UK higher education sector. It has clarified expectations, opened channels of dialogue, and given postdoctoral researchers a stronger voice in shaping the policies that affect them. These are significant achievements, demonstrating that cultural change is possible when funders, institutions, and researchers work together. Yet progress remains uneven: professional development continues to be undervalued, structural precarity persists, and the implementation of Concordat principles lacks visibility in many institutions. Without further action, these gaps risk eroding trust and undermining the sector’s ability to attract and retain research talent.

The confirmation of the REF 2029 framework provides a timely opportunity to accelerate change. The guidance makes clear that institutions must evidence how they value and support all those who contribute to research (REF, 2023, REF, 2025a). To meet this expectation, universities must embed stronger practices, particularly through systematic data collection and evidence-based evaluation, so that commitments move beyond statements of intent to drive meaningful and sustained improvement. Doing so will ensure the Concordat functions not merely as a set of principles, but as a practical framework for building a more inclusive, supportive, and high-performing research system in which postdoctoral researchers are recognised as central contributors.

## Data Availability

There are no data associated with this article.
